# Fracture Surface Evolution During Acidized Brine Injection in Calcareous Mudrocks

**DOI:** 10.1021/acsomega.3c00543

**Published:** 2023-05-16

**Authors:** Hasan Javed Khan, Ridha Al-Abdrabalnabi, Murtada Saleh Al-Jawad

**Affiliations:** Department of Petroleum Engineering, King Fahd University of Petroleum and Minerals, Dhahran, Eastern Province, Saudi Arabia 34464

## Abstract

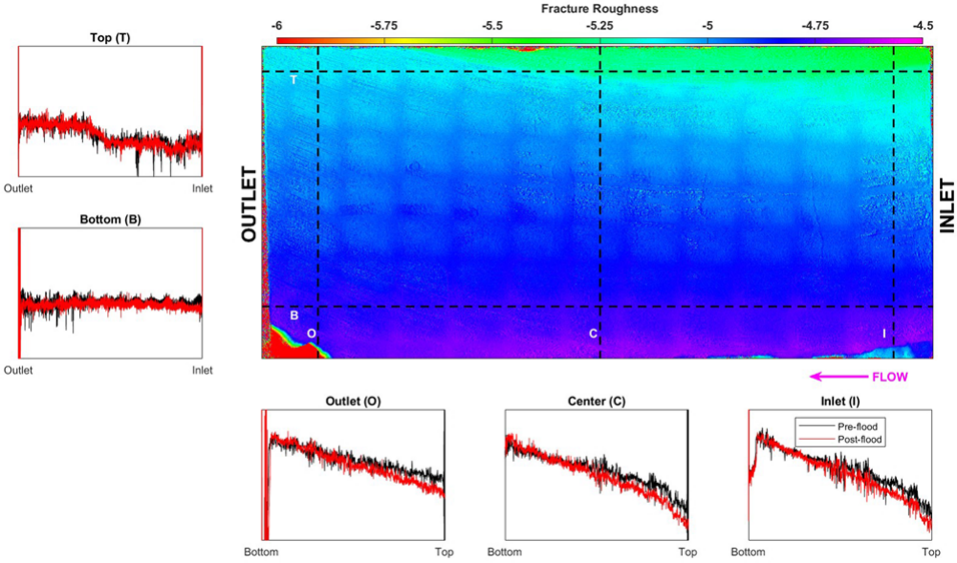

During hydraulic fracturing, the oxic hydraulic fracturing fluid physically and chemically alters the fracture surface and creates a “reaction-altered zone”. Recent work has shown that most of the physicochemical changes occur on the shale fracture surface, and the depth of reaction penetration is small over the course of shut-in time. In this work, we investigate the physicochemical evolution of a calcite-rich fracture surface during acidized brine injection in the presence of applied compressive stress. A calcite-rich Wolfcamp shale sample is selected, and a smooth fracture is generated. An acidized equilibrated brine is then injected for 16 h, and the pressure change is measured. A series of experimental measurements are done before and after the flood to note the change in physicochemical properties of the fracture. High resolution computed tomography scanning is conducted to observe the fracture aperture growth, which shows an increase of ∼8.3 μm during the course of injection. The fracture topography, observed using a surface roughness analyzer, is shown to be smoother after the injection. The calcite dissolution signature, i.e., surface stripping of calcite, is observed by X-ray fluorescence, and mass spectrometry of the timer-series of the effluent also points in the same direction. We conclude that mineral dissolution is the primary mechanism through which the fracture aperture is growing. The weakening of the fracture surface, along with the applied compressive stresses, promotes erosion of the surface generating fines which reduce the fracture conductivity during the course of injection. In this work, we also highlight the importance of rock mineralogy on the fracture evolution mechanism and determine the thickness of the “reaction altered” zone.

## 1 Introduction

Currently large volumes of oil and gas are commercially produced from low and ultralow permeability rocks (≪ 1 μDarcy). These formations have to be “activated” by hydraulic fracturing, which entails injecting a highly oxic, acidic hydraulic fracturing fluid at very high pressures inside the formation, which physically and chemically breaks the rock to generate high-flow pathways allowing hydrocarbons to be economically produced.^23^ A series of chemical reactions occur during the rock–fluid interaction including coupled mineral dissolution and precipitation, shale softening, fines migration, and wettability alteration.^19^ These reactions are highly dependent on the shale mineralogy, specifically the carbonate and clay content,^29^ and the hydraulic fracturing fluid composition. The general consensus is carbonates are dissolved with a reduction in pH, creating pore space on the fracture surface^4,12,15^ and generating dissolution-induced fines migration, which can plug up the pore space.^27,30^ Pyrite undergoes oxidative dissolution,^15^ which promotes H^+^ generation reducing the fluid pH^12,16^ and further dissolving the calcite, and the clay particles show great dependence on the ionic strength, which controls swelling, flocculation, and dispersion,^3,5,22^ thereby creating larger-sized mobile particles,^21^ which also block the effective flow pathways reducing the permeability of the rock^31,40^ and promote secondary clay mineralization due to presence of aqueous silica.^4^

Bratcher et al.^4^ conducted hydrothermal experiments on an organic rich mudstone under reservoir conditions in a gold reaction cell at varying pH and ionic strength. They found that calcite dissolution was independent of ionic strength at acidic pH, while feldspar dissolution was more dependent on the ionic strength and less on the fluid pH. No evidence of secondary mineralization of clay was observed. Similar experiments by Edgin et al.^9^ also yielded carbonate dissolution. A few of the other studies, conducted under advective conditions, in fractured^1,18^ and nonfractured^15^ shale cores have reported higher carbonate dissolution, increased fracture aperture growth, and more prominent etching patterns on the fracture surface. A recent study,^18^ conducted at room temperature and pressure and low flow rates, has shown that fines migration governs the fracture aperture growth. They saw the formulation of a weakened “reacted zone” due to mineral dissolution, which is then eroded with flow. This erosion not only strips the top damaged layer from the fracture surface increasing the fracture aperture but also increases the chance of the nonsoluble mobilized minerals to be trapped in a different spatial location reducing the fracture conductivity.^7,27,30,40^ Deng et al.^8^ had previously made similar observations at low flow rates that the soluble minerals were preferentially dissolved, which resulted in the development of a porous reacted zone, which was then eroded, opening up the fracture aperture. Gundogar et al.,^15^ in a similar experiment at constant low pressure, observed that the rock matrix softened due to mineral dissolution during core flooding in a nonfractured core, which ultimately resulted in permeability reduction under the compressive stresses. Others^6,34,35,39^ have shown that a difference in ionic strength between the host and injected fluid gives rise to (clay) fines being generated.

In reality, the shale rocks are under a high compressive stress and undergo periodic stress cycling during the injection period with high flow rates of fluid being injected. Furthermore, the injected and host fluid salinity is quite different,^10^ which can alter the fines generation and migration behavior. In this work, we experimentally test the impact of the former by suppressing the latter on the evolution of the fracture topography, the fracture surface mineralogy, and the thickness of the altered zone by injecting an acidized equilibrated brine at a high flow rate in a fractured calcite-rich shale. The manuscript is structured as follows: first, we describe the experimental methodology used, including sample preparation, acidized brine injection, and physical (fracture surface roughness measurement, X-ray scanning (μCT), and rock strength measurement) and chemical analysis (μXRF and effluent chemistry) techniques. The results for each of these experimental methods are displayed next followed by a comprehensive discussion on the impact of the compressive stresses on the thickness of the reacted zone.

## 2 Experimental Materials and Methods

**Figure 1:**
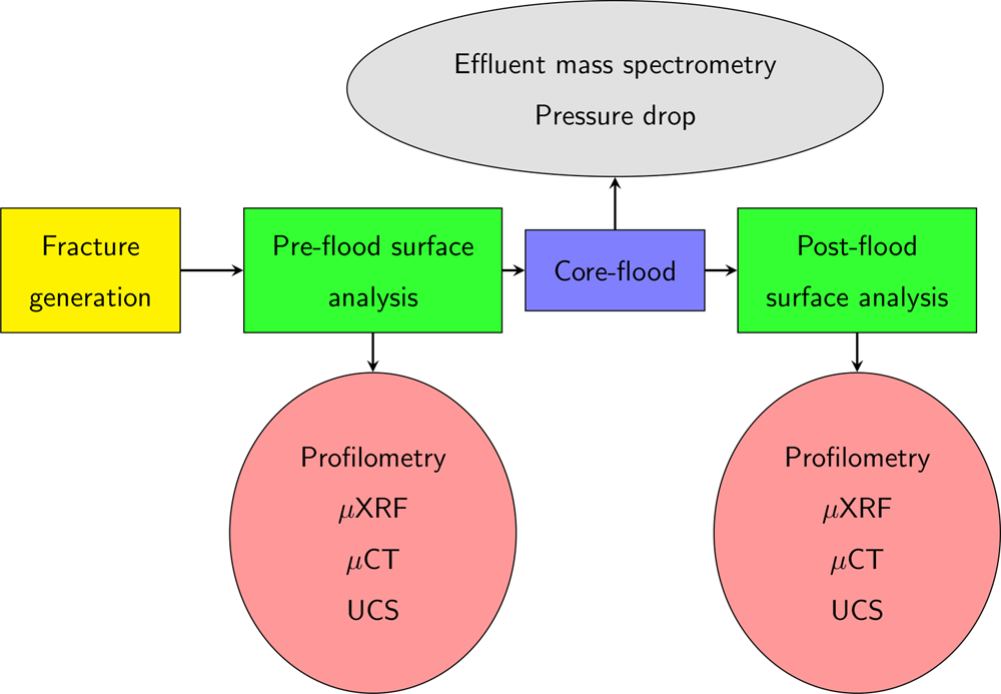
Experimental workflow. The rock sample is cut using a diamond saw to generate a smooth fracture. The fracture surface is chemically (μXRF) and physically (unaxial compressive strength (UCS) and profilometry) analyzed, and the fracture aperture is measured (μCT). The fracture is flooded with reactive brine during which a pressure drop is measured, and effluent samples are periodically collected for mass spectrometry. Postflood fracture surface and fracture aperture are physically and chemically analyzed, and the physicochemical changes in the fracture are noted.

The complete experimental workflow is presented in Figure 1. The shale sample is first fractured, and the two fracture surfaces are physicochemically characterized to determine the fracture topography, elemental distribution maps, rock strength, and fracture aperture. Reactive brine is then injected, and the pressure drop is measured while collecting the effluent. The fracture surface characterization is then repeated. This section details the experimental procedure.

### 2.1 Sample Preparation

A Wolfcamp shale core sample, 1.5-in. (37.5 mm) in diameter and 2.9-in. (72.5 mm) in length, was procured from Kocurek Industries Inc. (TX, USA). A small number of thin calcite-filled fractures at different orientations were observed on the core along with a single large calcite-filled fracture running along the length of the core. An artificial smooth fracture was created by dry cutting the core using a diamond-edged blade on the Mecatome ST310 circular saw by Presi (France). X-ray diffraction shows a mineral composition of 96.6% calcite and 3.4% quartz.

### 2.2 Acidized Brine Injection

#### 2.2.1 Brine Selection

An equilibrated brine recipe for Wolfcamp shale (subsurface samples) was designed by Khan et al.^18^ by reworking the produced water chemistry from the HFTS-1 well under the requirement of charge balance and nonreactivity. Brine with the same recipe (Table 1) was formulated and tested on the current samples by exposing small chips of the shale sample to the brine in the absence of air. The temporal evolution of the major cations (Na, Ca, Mg, Fe, Si, and K) was measured over 21 days using mass spectroscopy (ICP-MS), and no discernible change was observed. The equilibrated brine was titrated with 37% HCl until a pH of 2 was achieved to formulate an acidized brine that was used in the experiment. The equilibrated brine was acidized to ensure that the geochemical changes were occurring due to the acidity of the fluid rather than the brine composition.

**Table 1:** Equilibrated Brine Recipe Used in the Experiment^a^

salt	concentration (mg/L)
KCl	708
CaCl_2_	3330
MgCl_2_	950
NaCl	57833
NaNO_3_	41
Na_2_SO_4_	511
NaHCO_3_	249

a The recipe was generated from reworking the produced water chemistry under the conditions of non-reactivity and charge balance. Reproduced with permission from ref (18). Copyright 2022 American Chemical Society.

#### 2.2.2 Acid Flooding Setup and Workflow

**Figure 2:**
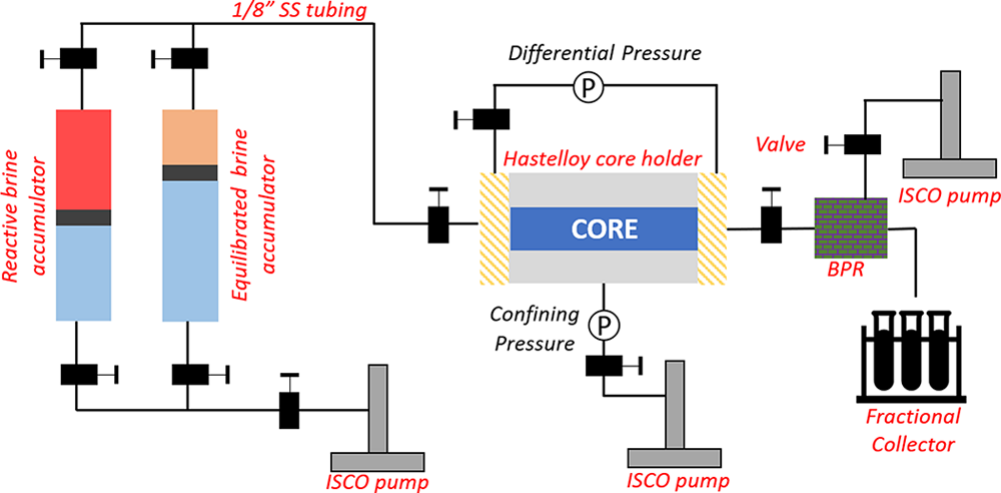
Acidized brine injection setup. The fractured core is placed in the core holder, which is connected at the upstream end to two accumulators (with reactive and equilibrated brine) in parallel and at the downstream end to the back-pressure regulator (BPR). The piston in the accumulator is operated by water via an ISCO pump. Confining pressure to the core and the pressure for the BPR are also provided by separate ISCO pumps. The effluent passes through the BPR and is collected by the fractional collector.

The fractured shale sample is closed along the fracture face and wrapped in heat-shrink tubing. The core is then put in a rubber sleeve and placed inside a Hastelloy coreholder. The coreholder is connected via stainless steel tubing to two accumulators through a manual three-way valve: one is filled with acidized brine (pH 2) and the other with the equilibrated brine (pH 7), which are operated by a flow pump. A differential pressure gauge records the pressure drop across the core. A back-pressure regulator set at 900 psi is placed on the outlet end of the coreholder which is then connected to a sample collector. An ISCO pump is used to provide a confining pressure of 1000 psi on to the rubber sleeve. The complete acidized brine injection setup is shown in Figure 2.

The flooding experiment starts by injecting the fractured core sample with the equilibrated brine (pH 7) for 24 h to ensure complete saturation of fracture-adjacent zone in the core. The pressure drop at varying flow rates (1, 2, and 4 mL/h) is also measured to calculate the fracture conductivity, which is defined as the fracture permeability (*k*_f_) times the fracture width (*w*_f_). Seepage characteristics of the rock are an important parameter during calculation of the fracture conductivity in fractured conventional porous media.^24,25^ In the current experiment, the near-fracture zone was saturated and coupled with the low permeability of the shale medium; the shale seepage was not considered to be of high importance in the fracture conductivity calculation.

The three-way valve is then flipped manually and the acidized brine injection (pH 2) is started at an injection rate of 0.1 mL/min (6 mL/h). The acidized brine is injected for a total of 16 h. The pressure drop is continuously recorded, and the fluid effluent is collected every hour. The outlet dead volume of the system is calculated to be 6 mL. After the acidized brine injection is complete, the equilibrated brine is injected to flush out the nonreacted acidized brine and stop any reaction happening in the core. The pressure drop at varying flow rates is again measured to calculate the new postinjection fracture conductivity.

### 2.3 Fracture Surface Roughness

The surface roughness of the exposed fracture face is measured using a surface roughness analyzer,^11^ which moves a laser source over the fracture surface placed on an elevated platform and measures the time of reflection. This correlates directly to the surface elevation at the measurement point and, combined with the other measurements over the 2D surface, gives the surface roughness. Each of the fracture faces (HK1 and HK2) is scanned before and after core flooding at a lateral spatial resolution of ∼10 μm and a depth resolution of ∼1 μm using a Surface Roughness Analyzer by KRÜSS GmbH (Germany) present at the College of Petroleum and Geosciences, KFUPM, KSA. The analyzer is customized with a larger scanning base (Thorlabs 1530F/M aluminum breadboard by Thorlabs, NJ, USA), 150 mm × 300 mm, to allow longer samples to be scanned in one go to reduce stitching artifacts. The generated data are analyzed using MountainsMap to measure the change in the fracture surface during the acidized brine injection.

### 2.4 Microcomputed Tomography Scanning

A preflood and postflood computed tomographical (CT) scan is conducted using the HeliScan MicroCT (ThermoFisher Scientific, USA). The dried fractured sample is wrapped in heat shrink tubing (to maintain the rock position) and helically scanned at a voxel resolution of 16.2 μm using 4420 projections in 3 h and with scanning parameters of 65 mA current, 95 kV voltage, and 1.1 s exposure time. The resultant volume is sliced into 4262 slices of a 16-bit image. Therefore, each slice has a voxel resolution of 16.2 μm, and the separation between each slice is also 16.2 μm.

**Figure 3:**
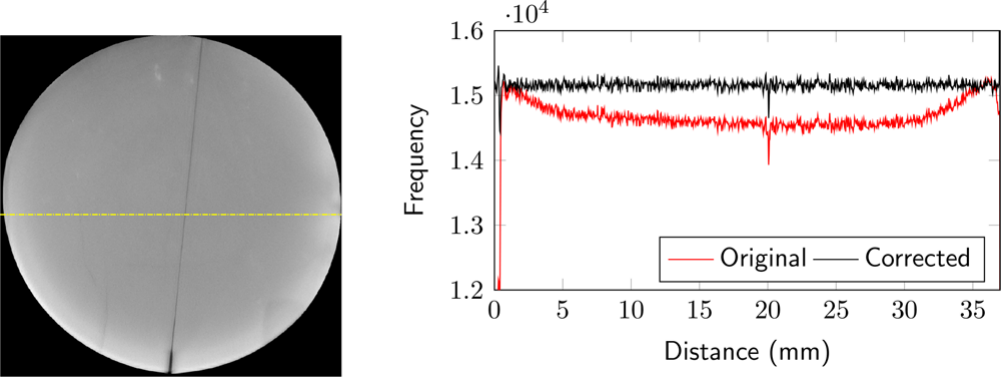
Micro-CT output acquired at a resolution of 16.2 μm showing the effect of beam hardening. The profile generated along the yellow dashed line shows the characteristic behavior of beam hardening (red), which is corrected (black) before segmentation.

The output from the micro-CT is displayed and analyzed in Fiji-ImageJ^32^ and MATLAB.^26^ The image stack is first corrected to reduce the effect of beam hardening (Figure 3). The images are then registered, aligned, and cropped to remove the heat-shrink tubing surrounding the core. The stack is then passed through a median filter (radius 5) to clean and despeckle the image. A local threshold is then applied to segment the solid phase (core), void space (fracture volume), and the rest (outside the core) individually.

### 2.5 Rock Strength

The shale sample’s unconfined compressive strength (UCS) is measured by the scratch test method before and after the acidized brine injection using the Wombat Scratch Test Machine by Epslog Engineering (Belgium) located in the College of Petroleum and Geosciences, KFUPM, KSA. The scratch test is a quick, semi-nondestructive method that utilizes a scratching tool to indent the rock surface up to 1 mm and can be used to estimate the rock’s geomechanical properties, including the fracture toughness^20^ (i.e., the resistance to fracture propagation) and unconfined compressive strength (UCS;^14^ i.e., the maximum axial compressive stress that shale can bear under zero confining stress). The scratch test provides the UCS value by correlating it to the rock specific energy, which are measured from the applied shear and normal forces.^14^ Previous studies^18^ have proposed that the fracture surface weakens due to mineral dissolution during acidized brine injection.

The test requires a smooth flat surface; therefore the first step is to shave the curved surface and create a smooth channel across which the measurement is conducted. Then the indent is created on the surface and the indenter is dragged along the length of the sample while measuring the force required to make the scratch. The process is then repeated with a deeper indent, i.e., farther away from the original surface. For each indent depth, the rock strength is calculated, resulting in the UCS profile as a function of distance away from the fracture surface.

Since the fracture surface is damaged during the scratch test, the outer curved surface of the core is used for the prereaction UCS measurement, and the inner fracture surface is used for the postreaction UCS measurement on the acidic brine flooded core. The semi-nondestructive implies that the rock surface is damaged, but the post-test rock sample remains intact for other destructive or nondestructive tests.

### 2.6 Chemical Analysis

#### 2.6.1 Micro-X-ray Fluorescence

Fracture surface elemental 2D maps are generated using the M4 Tornado μXRF spectrometer by Bruker Corporation (USA) present at the College of Petroleum and Geosciences, KFUPM, KSA. The spectrometer uses a small spot size to generate a high resolution (∼60 μm) surface map of the two fracture surfaces before and after acidized brine injection. The difference between the 2D elemental maps generated before and after the acidized brine injection shows the change in mineral composition on the fracture surface, which can be attributed to the acidized brine injection.

#### 2.6.2 Effluent Analysis

The effluent is collected every hour, and the major cations are analyzed in an inductively coupled plasma optical emission spectrometer (ICP-OES).

## 3 Results

### 3.1 Fracture Aperture Evolution

**Figure 4:**
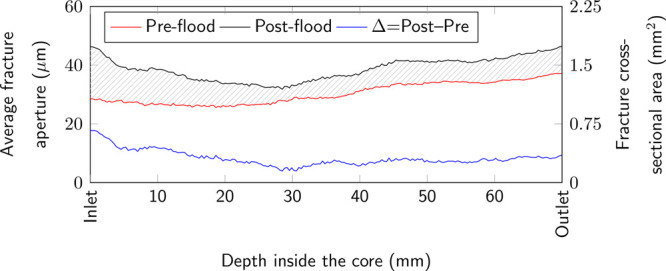
Average fracture aperture increased during acidized brine injection from 30.3 to 38.6 μm with the largest average fracture aperture change observed at the inlet.

The segmented image gives an independent voxel value to each phase: 0, solid; 1, void; and 2, outside. The total number of voxels in the fracture phase in each slice are counted and multiplied by the voxel area (16.2^2^ μm^2^). This results in the fracture cross-sectional area per slice, which is then divided by the fracture width (37.5 mm) to get the average fracture aperture in each slice. Figure 4 plots the spatial distribution of the average fracture aperture and fracture cross-sectional area per slice along the length of the core for the pre- and postflood core. The difference between the pre- and postflood curves shows the change in fracture aperture at each depth and is highlighted in a gray line pattern and is also plotted separately in blue.

The preflood fracture aperture is quite uniform for the first half of the core (∼26.5 μm), before it increases at the outlet to ∼37 μm. The sharp increase at the outlet is potentially due to chipping of the side of the core during the fracture creation, which is also evident in surface topography scans shown later (Section 3.3).

### 3.2 Fracture Conductivity

**Figure 5:**
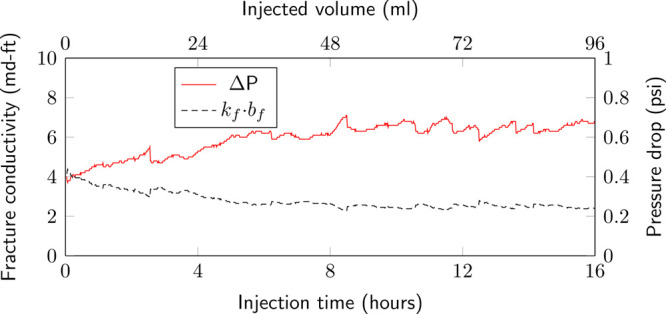
A continuous rise in pressure (solid red line) observed during the acid injection phase, resulting in a 3-fold increase in pressure and therefore decrease in the fracture conductivity (dashed black line).

The pressure drop across the length of the fracture is measured before, during, and after acidized brine injection (Figure 2) and is used to calculate the fracture conductivity over time (Figure 5) using Darcy’s law:
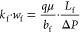
1where *k*_f_·*w*_f_ is the fracture conductivity, μ is the fluid viscosity, *q* is the injection rate, *b*_f_ is the fracture height, *L*_f_ is the fracture length, and Δ*P* is the pressure drop across the core.

The pre- and postflood fracture conductivity is measured with the equilibrated brine injected at multiple flow rates (1, 2, and 4 mL/min), resulting in fracture conductivity measurements of 3.82 and 5.28 md-ft, respectively. Interestingly, the pressure drop measured during the acidized brine injection shows an increasing trend with the volume of injectate. A low pressure drop (0.40 psi) is observed at the start of injection, which corresponds to a fracture conductivity of 4.05 md-ft. By the end of the experiment, the pressure drop has increased to 0.68 psi or a fracture conductivity of 2.38 md-ft.

### 3.3 Fracture Surface Evolution

#### 3.3.1 Fracture Topography

**Figure 6:**
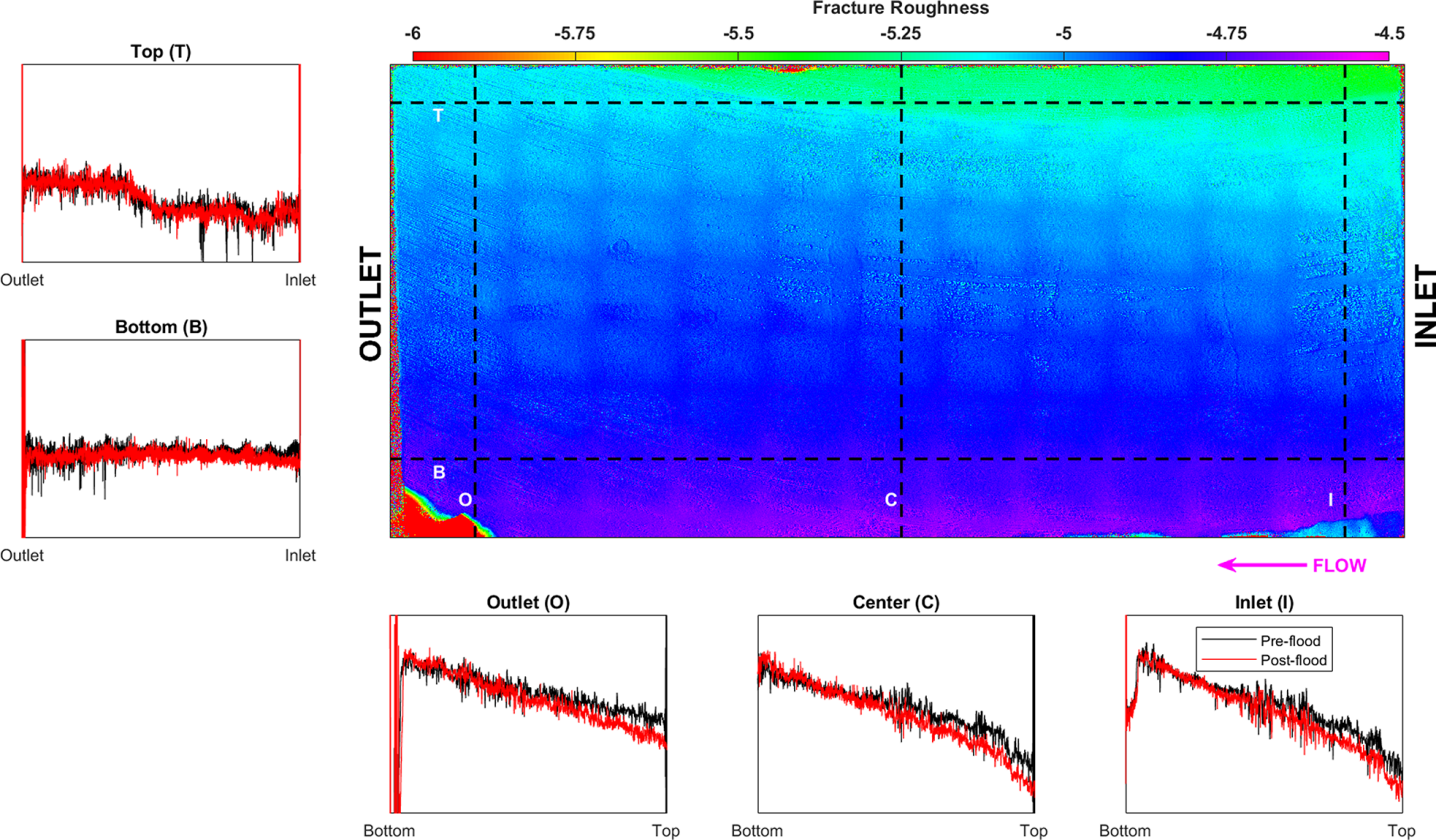
Surface roughness analysis done on the pre- and postflood H2 fracture surface. The color intensity map represents the distance of the preflood fracture surface to the sensor and shows the fracture to be dipping toward the top side. The fluid is injected from the right. Five profiles are generated at the top (T), bottom (B), inlet (I), center (C), and outlet (O) of the fracture for the preflood (black) and postflood (red) scan. The bottom-outlet end is aligned to show the relative change in the surface profile during acidized brine injection.

Pre- and postflood fracture surface roughness is determined using a surface roughness analyzer. The generated topographical map (Figure 6) shows the distance of the fracture surface from the detector at each location at a resolution of ∼10 μm. The bright colors represent a higher elevation, and darker colors represent a lower elevation. The smooth fracture is dipping toward the upper part of the figure with a depressed segment at the upper part of the figure. A dark gridded pattern is also evident, which was later found to be caused by the measurement sensor being misaligned.

**Figure 7:**
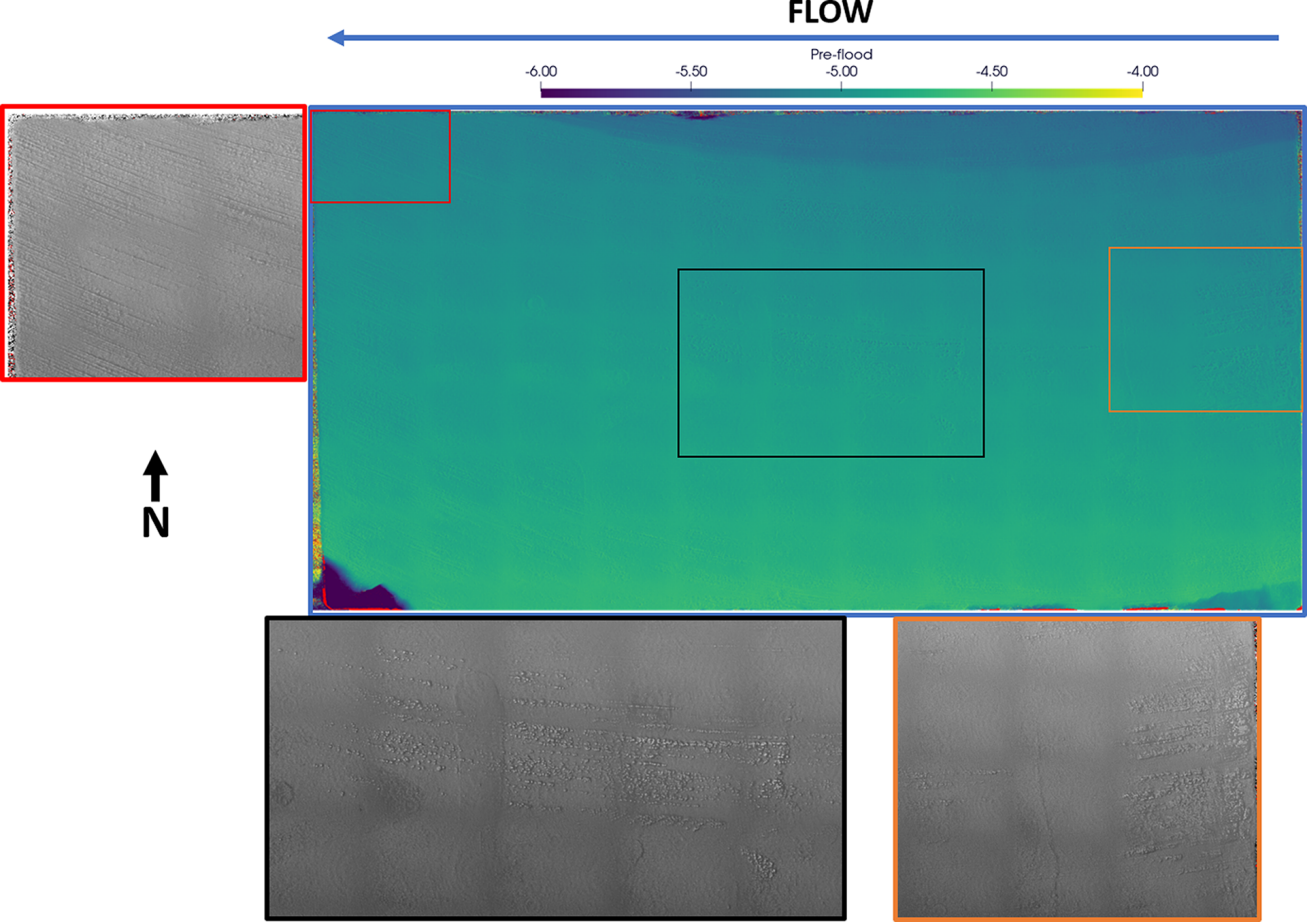
Surface profilometry scan on the fracture surface H2 before injecting the acidized brine from left to right. The distance from the surface to the detector is measured and is presented as a color map (blue): bright for near the detector and dark for away from the detector. The smooth fracture is dipping toward the north position with chips observed in the southwest and southeast corners. Zoom-in regions at the inlet (orange), center (black), and outlet (red) are presented in grayscale where the bright color shows depressions on the surface.

**Figure 8:**
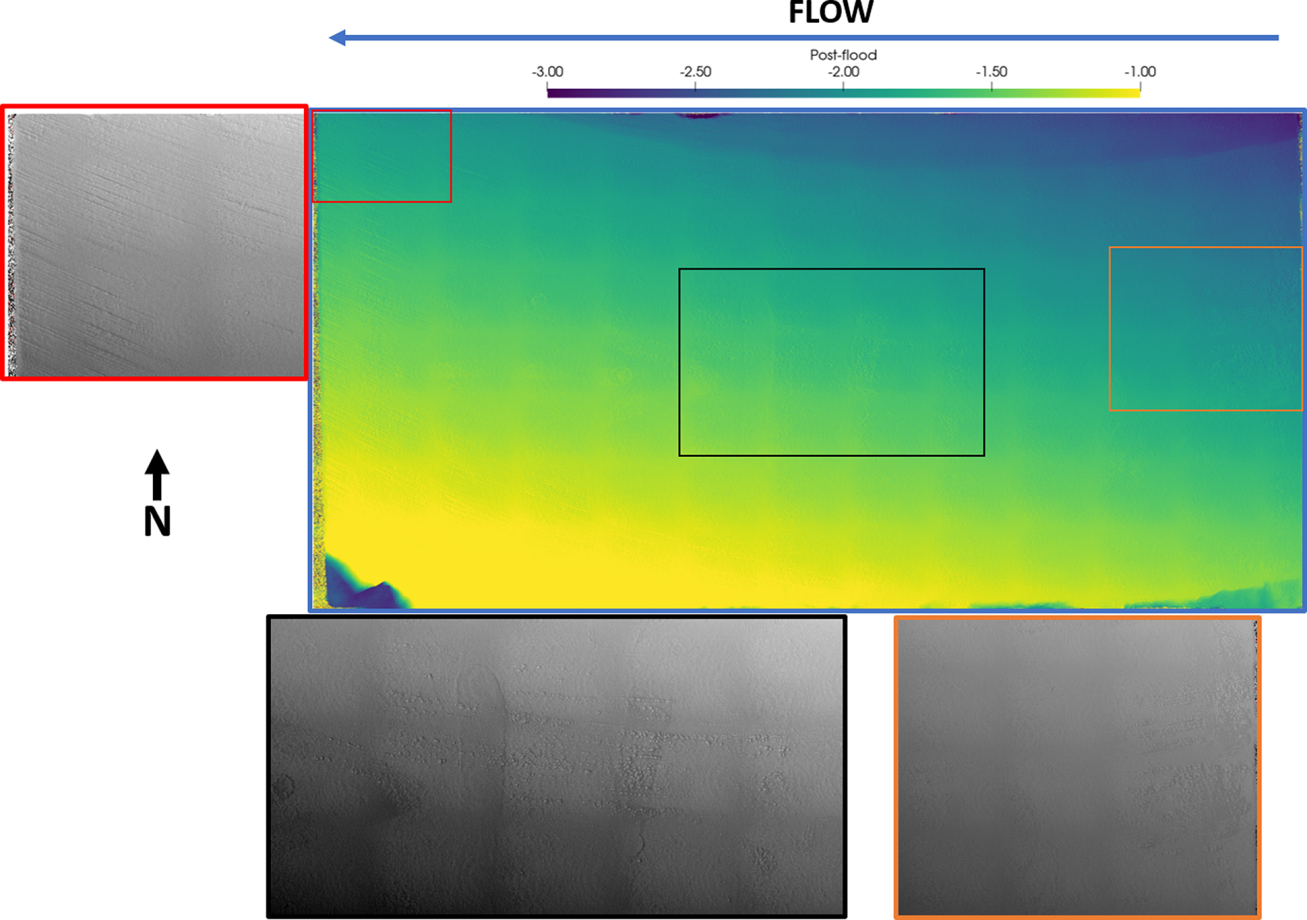
Surface profilometry scan on the fracture surface H2 after injecting the acidized brine from left to right. The distance from the surface to the detector is measured and is presented as a color map (blue): bright for near the detector and dark for away from the detector. The smooth fracture is dipping in the north–northeast position with chips observed in the southwest and southeast corners. This is indicative of rock dissolution near the inlet face, specifically in the northest position. Zoom-in regions at the inlet (orange), center (black), and outlet (red) are presented in grayscale where the bright color shows depressions on the surface. The fracture surface has smoothed out with surface abrasions reduced.

The preflood scan (Figure 7) shows a lot of evidence of small pits at the inlet (orange) and center (black) of the fracture (generally aligned in middle of the fracture), a fracture running across the direction of flow at the inlet (orange), and cutting abrasion marks at the outlet (red). The same spatial locations observed in the postflood scan (Figure 8) show stark differences. The small pits observed at the inlet (orange) of the fracture see the most significant change, with the pits being obscured due to reduction of the surrounding area to the same elevation as the pit. The fracture at this point is also smoothed and visible in the postflood scan. Smaller changes are seen in the center (black) of the fracture where multiple pits have been obscured and/or the depth has been reduced, though multiple pits are still evident at this point. The abrasion marks have also been smoothed out with less abrasion visible at the outlet (red).

The difference between the pre- and postflood topographical maps can also be used to quantify the evolution of the fracture surface due to the reactive brine injection. Figure 6 shows five associated plots, which are the profiles along the lines drawn in different areas of the topographical map: top (T), bottom (B), inlet (I), center (C), and outlet (O). Each plot shows the surface profile for the preflood (black) and postflood (red), which have been aligned to be zero at the bottom (Figure 6I,C,O) and outlet (Figure 6T,B) values for each curve.

Generally, the fracture surface is quite uniform along the direction of flow (Figure 6T,B black). Minor variations in the preflood profile are observed at the top and bottom part of the fracture; the top profile shows the depressed segment observed in the topographical map in the upper part of the figure. The postflood profiles show a slight decrease at the inlet (Figure 6T,B red) showing evidence of matrix removal. Furthermore, the postflood profiles show less undulations, implying smoothing of the fracture surface.

The preflood profiles generated across the direction of flow (Figure 6I,C,O black) show the bottom part to have a higher elevation than the top part. Little variations are observed all along the length of the core with a sharp decline observed at the depressed segment. All of the postflood profiles (Figure 6I,C,O red) show a consistently lower value, with more pronounced change at the top part of the fracture.

#### Fracture Toughness

**Figure 9:**
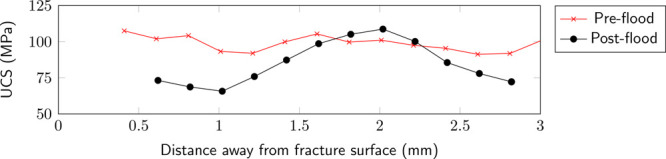
Pre- and postflood rock strength as a function of distance away from fracture surface.

A scratch test conducted on the pre- and postflood surfaces shows that the unconfined compressive strength reduces from 101.5 to 86.1 MPa over the course of injection. As mentioned before (Section 2.5), since the test is semidestructive, the preflood scratch test is conducted on the outer curved surface of the sample, and the postflood scratch test is done on the reacted fracture surface. The rock strength on the postreaction surface changes as a function of the distance away from the fracture surface (Figure 9), which is representative of the evolution of the fracture due to the acidized brine injection and the generation of the reacted zone. The near fracture face (postflood) shows the highest change in rock strength with UCS as low as 65 MPa within the first millimeter, which then increases to the preflood value of around 2 mm from the fracture surface. The reacted altered zone can therefore be estimated to be between 1 and 1.5 mm from the fracture surface. A sharp decline in the strength is then observed, which coincides with the presence of a thin calcite-filled fracture present in the rock.

### 3.4 Chemical Analysis

#### 3.4.1 Micro X-ray Fluorescence

Micro-X-ray fluoroscope scans are conducted on the fracture surface before and after acidized brine injection. The fracture surface, which was generated by sawing the core in half, is predominantely Ca (Table 2) with a minor presence of Fe, Si, Mg, and Na. Si, K, and Na show increases of greater than 20%, which can be attributed to the exposure of plagioclase and K-feldspar from the underneath layers. Plagioclase (specifically albite) and K-feldspar are commonly found in multiple facies of the Wolfcamp shale.^2,28^

**Table 2:** Elemental Composition of the Fracture Surface Determined through μXRF before and after the Acidized Brine Injection^a^

element	K	Na	Ca	Mg	S	Si	Fe
preflood (wt %)	0.16	0.27	97.11	0.43	0.23	1.27	0.50
postflood (wt %)	0.11	0.36	96.81	0.40	0.22	1.56	0.52

a Calcite dissolution is evident from the reduction in Ca content.

**Figure 10:**
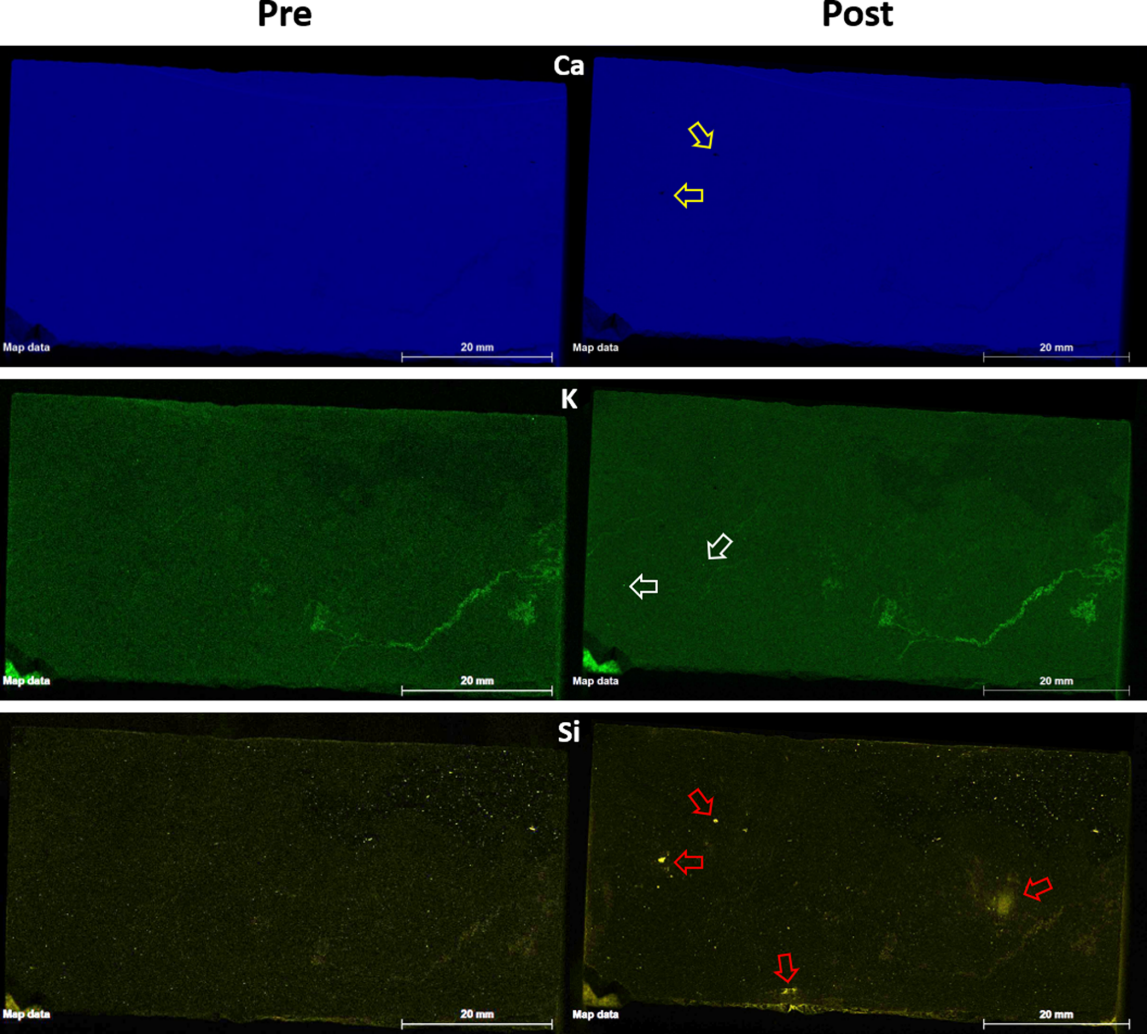
Micro-XRF of the fracture surface before (left) and after (right) injection. Injection occurred from right to left.

Composite elemental maps of the fracture surface are presented in Figure 10. Ca is found all over the fracture surface with sparse sporadic distributions of Fe and Si. K-feldspar is present filled in a vein in the lower right corner in the preflood scan (Figure 10, left column). The postflood scans (Figure 10, right column) show a similar behavior with a dominant display of Ca and sporadic distribution of Si and Fe. More empty zones are observed in the Ca map (yellow arrow) compared to the preflood scan. These show up as bright spots on the Si map (red arrow), implying either removal/erosion of Ca from the fracture surface or entrapment of Si fines on the fracture surface. Interestingly, most of the changes between the pre- and postflood μXRF scans are observed at the outlet end (left side) of the core.

#### 3.4.2 Effluent Analysis

**Figure 11:**
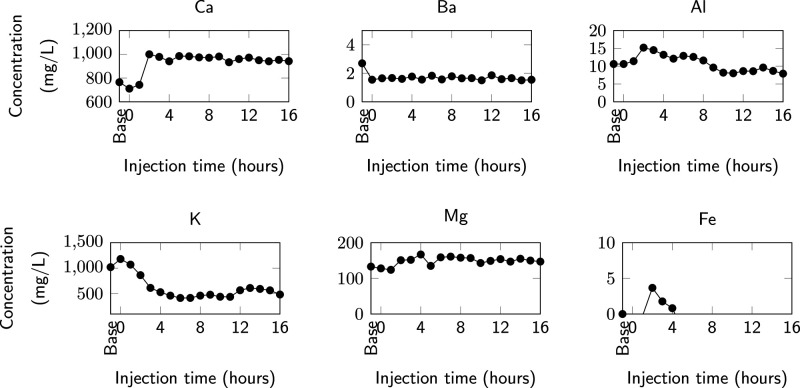
Time series of the geochemistry of the effluent fluid collected every hour. The outlet dead volume is 6 mL (= 1 h injection). Little to no change is observed in Mg and Ba. Ca increases by ∼200 mg/L due to calcite dissolution by the acidic brine. Al shows a small rise before it settles down later during the injection. K shows a consistent decrease and reduces by over 50% during the course of the injection. A small spike in Fe is observed as the first reacted effluent is collected.

The dead volume of the system, i.e., the volume between the core and the fractional collector, is measured to be 6 mL. Since the experiment utilizes an injection rate of 0.1 mL/min or 6 mL/h and the sample is collected every hour, we can readily determine that the effluent collected at 1-h postinjection would be representative of the equilibrated brine and not of the injected reactive brine. The reactive fluid front has not reached the fractional collector at 1 h. The effluent is analyzed in an ICP-OES to determine the major cations present in the fluid and is presented in Figure 11.

Of the six elements measured, the most drastic change is observed in Ca and K. Ca shows an increase of ∼25%, which continues for the duration of the experiment. This roughly translates to ∼200 mg/L or 5 mM Ca being added to the fluid due to its interaction with the fracture surface. K on the other hand reduces by ∼50% to 500 mg/L (or 12.8 mM). Fe is not present in the base fluid and represents itself at the early breakthrough, after which it is not observed in solution anymore. The Fe is potentially produced from the dissolution of acid-soluble iron compounds^17,38^ or can be due to rusting in the stainless-steel tubing, which connects the accumulator to the core holder. Xiong et al.^38^ also observed this sharp increase and decrease in Fe concentration during the reactive flood in a carbonate-rich Wolfcamp shale core. For Al, though exhibiting ±50% variation, the absolute value is very small, making the effect negligible. Mg also shows a ∼25% increase during the course of injection with a final concentration of 6.1 mM. Ba is consistent throughout the injection duration.

## 4 Discussion

The experimental analysis over the course of injection has resulted in four main observations:

1. The cross-sectional area of the rock sample at the inlet is reduced.
2. The fracture surface has smoothed.
3. The fracture surface gets weaker and more prone to shear failure, indicating the development of the reacted zone.
4. New Si “growths” are observed on the postreaction fracture surface.

The average fracture aperture is measured using μCT scanning and shows a 30% increase (from 30.3 to 38.6 μm) over the course of injection (Table 3). Based on the effluent analysis (Figure 11), carbonate dissolution is expected to be the most prominent cause of this fracture aperture growth. Fracture topography analysis on the pre- and postflood fracture surface shows similar results: the fracture surface has become tapered with the inlet at a slightly lower elevation compared to the outlet (Figure 6T,B). The same is observed in the fracture aperture growth, where a larger increase is observed at the inlet (Figure 4). All of these point to the fact that carbonate dissolution is more prominent at the inlet, where fresh reactive fluid is more readily available.

**Table 3:** Pre- and Post-Reaction Experimental Results Including Average Fracture Aperture, Fracture Conductivity, and Unconfined Compressive Strength

core	average fracture	fracture	unconfined compressive
status	aperture (μm)	conductivity (md-ft)	strength (MPa)
prereaction	30.3	3.82	101.5
postreaction	38.6	5.28	86.1

The chemical reaction on the surface has also resulted in smoothing of the fracture surface. The smaller undulations present in the preflood scan on the fracture surface (Figure 6) have been dissolved. Interestingly, this change is observed across the length of the core and not only focused at the inlet end of the core. The fracture surface also is observed to be weaker due to the rock-fluid interaction, with a 15% loss in compressive strength observed (Table 3). Khan et al.^18^ had previously proposed that the calcite dissolution on the shale fracture surface will cause the surface to become “weak,” generating the reacted zone, and the succeeding fluid injection will result in erosion of the nonsoluble minerals and increase the fracture aperture. Prior to that, Deng et al.^8^ had also observed a porosity increase in the reacted zone in a calcite-rich shale, making it weak. Shovkun and Espinoza^33^ also showed that dissolution of calcite from the fracture surface would result in shear failure of the rock, which can result in erosion of the fracture surface. The fracture toughness measurement (Table 3) indicates the reacted zone thickness to be 1–1.5 mm. Khan et al.^18^ quantified the thickness of the reacted zone thickness as 0.2–0.8 mm (outlet to inlet) from their reactive transport model for a 116 fracture volume of injectate pumped. For the current study, which has an 8× larger calcite concentration, a total 1270 fracture volume of injectate (11× larger) was injected. Since the calcite concentration would reduce the thickness of the reacted zone and the volume of injectate will increase it, we can expect the thickness of the reacted zone to be around 0.3 at the outlet to 1.1 mm at the inlet. Therefore, the measured thickness of the zone (1–1.5 mm) indicates other reactive processes happening at the fracture/fluid interface, which are not being accounted for in their reactive transport model.

**Figure 12:**
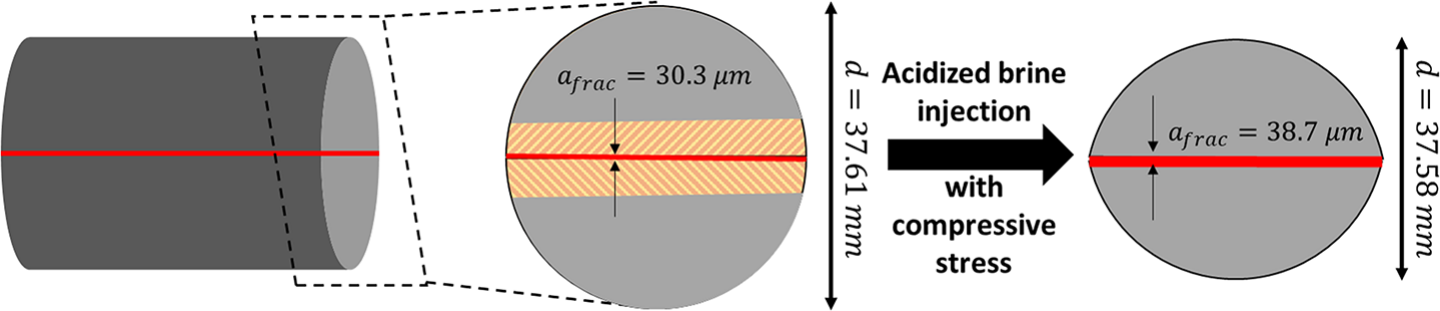
Core compression process

The fracture conductivity shows a reduction by 40%, from 4.05 to 2.38 md-ft (Figure 5), over the duration of injection. This is counterintuitive to the expectation that an increase in fracture aperture should result in an increase in the fracture conductivity. But considering the weakening of the fracture surface and the applied compressive stresses (Figure 12), the probability of nonsoluble fines being generated increases, which can result in a reduction of the fracture conductivity, even though the overall fracture aperture is increasing in all parts of the fracture. Tripathi and Pournik^36^ and Weldu Teklu et al.^37^ had similar observations when interacting strong and diluted HCl, respectively, with calcite-rich shales. The fracture conductivity change observed during the injection was opposite to what the pre- and postflood fracture conductivity measurements have shown, which show an increase in the conductivity (Table 3). The pre- and postflood conductivity measurements were done at a significantly higher flow rate (1–4 mL/min compared to 0.1 mL/min for the acidized brine injection), which can potentially release the entrapped fines at the constrictions inside the fracture.

Previous work,^18^ conducted in the absence of confining pressure, showed fracture widening by up to 55 μm or 8.5% during the first 16 h of reactive brine flooding. During this time period, a total of 34.5 fracture volume of brine was injected, resulting in an average change of 0.25% per fracture volume of injectate. The current study had an initial fracture volume of 0.0756 cm^3^, and a total 1270 fracture volume of the reactive brine is injected. This resulted in the fracture volume changing by ∼27%, which gives an average change of of 0.021% per fracture volume, which is an order of magnitude lower than in the absence of confining pressure. Considering that the injection rate is 10× greater in the current study (i.e., greater chances of erosion) but the calcite composition is an order of magnitude higher (i.e., more injectate required for same “thickness” of calcite to be dissolved), the approximate fracture volume change per fracture volume of reactive brine injected is still low in the presence of the confining pressure. Therefore, we can conclude that the presence of confining pressure, which is the case for actual hydraulic fractures in a shale reservoir, will result in a smaller increase of the fracture aperture at low to medium flow rates.

Even though calcite dissolution is the dominant reaction during the fracture-brine interaction, Ca is still the most prominent element on the fracture surface. Dissolution pits and erosion behavior are observed in the μXRF Ca postflood scans (Figure 10), indicating removal of Ca from the top layer. The high calcite concentration also indicates that calcite dissolution is not channelized; rather face dissolution would occur on the fracture/fluid interface.^13^ This exposes nonsoluble minerals (e.g., quartz) onto the surface which exhibit as Si “growths” on the fracture surface. More of these growths are present near the outlet end, which can potentially be due to fast removal (erosion and dissolution) of the rock material at the inlet, thereby increasing the dissolution-induced fines migration.

Considering 16 h of acidic brine injection (*q* = 0.1 mL/min) and 5 mM of Ca being added to the brine (Figure 11) due to the reactive interactions, roughly 0.48 mmol of Ca was dissolved from the fracture surface. This equates to 0.048 g or 0.0177 cm^3^ of calcite being removed from the fracture surface. X-ray crystallography (XRD) of the sample showed a high calcite content of 96.6%. As previously mentioned, the high calcite content ensures face dissolution, which implies a uniform rate of dissolution across the fracture surface. For the scenario above, this equals ∼6.8 μm of rock layer being removed from the fracture surface during the acidic brine injection. The average fracture aperture change experimentally observed due to injection is (38.6 – 30.3 =) 8.3 μm. Considering the heavy handedness of the assumptions, this 20% difference is not large enough to conclusively indicate the presence of a secondary solid removal mechanism, such as fines migration. The phenomenon might have been present, albeit on a smaller scale, and therefore would have been a lesser influence.

Summing up the previous conclusions for the condition where confining pressure is present, we have a smaller increase in the fracture and a larger increase in the reacted zone. Both of these contribute positively to the system’s flow properties. Coupling these with the presence of the fines migration phenomenon, we can potentially see the fines being trapped in the reacted zone hindering the fracture conductivity. Furthermore, some of the fines can also be trapped in the constrictions in the fracture, which can result in the overall decline in the fracture conductivity that is observed in this experiment.

## 5 Conclusion

Here, we have reported an experimental study designed to investigate the phyiscochemical evolution of a calcite-rich fracture surface during acidized brine injection under compressive stresses. Using X-ray computed tomography, surface roughness profilometry, a scratch test, and pressure logging, we tracked the physical change in the fracture aperture and the fracture topography and have quantified the thickness of the “reaction altered” zone based on the evolution of the fracture surface’s mechanical properties. By using X-ray fluorescence and mass spectrometry, we track the chemical evolution of the fracture surface and highlight that the main reaction occurring on the fracture surface is calcite dissolution. Major observations during the course of the experiment include (1) an increase in the fracture aperture with the largest change observed at the inlet, (2) smoothing of the fracture surface, (3) reduction in the fracture conductivity during the course of injection, (4) preferential dissolution of calcite, and (5) weakening of the fracture surface, indicating a reacted zone thickness of 1–1.5 mm. Simple back-of-the-envelope calculations show that fines migration is present but is not very dominant. The majority of the fracture aperture change is happening due to calcite dissolution, but this change is small when compared to the condition of no confining pressure. The thickness of the reacted zone is larger in this case, but this zone is more prone to fines generation and migration (due to shear weakening) and also prone to fines entrapment due to the presence of permeable pathways. Therefore, the combined effect can effectively reduce the fracture conductivity over time. These results are in agreement with the chemomechanical evolution idea proposed by Khan et al.^18^ and Deng et al.^7^ and show that this chemomechanical process is more prominent in the absence of confining pressure. These results also highlight the significance of fines migration and the importance of accurate quantification of the compressive stresses when estimating the hydrodynamic impact of unpropped hydraulic fractures in shales.
